# Parental Attitudes, Intentions, Decisions, and Psychological Wellbeing Regarding COVID-19 Vaccination: Preschool, School-Age, and Adolescent Caregivers

**DOI:** 10.3390/vaccines10122114

**Published:** 2022-12-10

**Authors:** Liang-Jen Wang, Kuang-Che Kou, Kuo-Shu Tang, Yu Lee, Yi-Chun Chen, Mao-Hung Lo, Ing-Kit Lee, Seng-Kee Chuah, Chien-Te Lee, Chia-Te Kung, Chih-Chi Wang, Shao-Ju Chien

**Affiliations:** 1 Department of Child and Adolescent Psychiatry, Kaohsiung Chang Gung Memorial Hospital and Chang Gung University College of Medicine, Kaohsiung 83301, Taiwan; 2 Division of Pediatric Infection, Kaohsiung Chang Gung Memorial Hospital, Kaohsiung 83301, Taiwan; 3 Division of Pediatric Emergency, Kaohsiung Chang Gung Memorial Hospital, Kaohsiung 83301, Taiwan; 4 Department of Psychiatry, Kaohsiung Chang Gung Memorial Hospital and Chang Gung University College of Medicine, Kaohsiung 83301, Taiwan; 5 Division of Infectious Disease, Department of Internal Medicine, Kaohsiung Chang Gung Memorial Hospital and Chang Gung University College of Medicine, Kaohsiung 83301, Taiwan; 6 Division of Pediatric Cardiology, Kaohsiung Chang Gung Memorial Hospital and Chang Gung University College of Medicine, Kaohsiung 83301, Taiwan; 7 Division of Hepato-Gastroenterology, Department of Internal Medicine, Kaohsiung Chang Gung Memorial Hospital and Chang Gung University College of Medicine, Kaohsiung 83301, Taiwan; 8 Department of Nephrology, Kaohsiung Chang Gung Memorial Hospital and Chang Gung University College of Medicine, Kaohsiung 83301, Taiwan; 9 Department of Emergency, Kaohsiung Chang Gung Memorial Hospital and Chang Gung University College of Medicine, Kaohsiung 83301, Taiwan; 10 Division of General Surgery, Department of Surgery, Kaohsiung Chang Gung Memorial Hospital and Chang Gung University College of Medicine, Kaohsiung 83301, Taiwan

**Keywords:** psychosomatic, infectious disease, depression, SARS-CoV-2, vaccine

## Abstract

The vaccination of all children may be one of the most important public health measures for preventing a wider spread of severe acute respiratory syndrome coronavirus 2 (SARS-CoV-2) infection in the community. Therefore, the purpose of this study was to investigate the attitude, intention, decision making, and psychological well-being among the caregivers of children who received SARS-CoV-2 vaccination in Taiwan. The caregivers of children (98 preschool children, 191 school-age children, and 154 adolescents) who received COVID-19 vaccination were invited to fill in the following questionnaires: Adopting Self-Protective Behavior Scale, Drivers of COVID-19 Vaccination Acceptance Scale, Impact of Event Scale, Chinese Health Questionnaire, and Parental Bonding Instrument. Compared to the caregivers of adolescents, the caregivers of preschool children exhibited more protective behaviors toward the COVID-19 pandemic. The caregivers of preschool children also displayed a higher emotional impact than those of adolescents and took a greater interest in the family’s opinion about vaccination. Finally, we found that COVID-19 ideological invasion and protective parenting style were significantly related to the prevalence of mental illness among caregivers. The results of this study can be used as an important reference for vaccination health care and policy formulation for adolescents with regard to COVID-19.

## 1. Introduction

The outbreak of coronavirus disease 2019 (COVID-19) continues to affect people all over the world [1,2]. Taiwan’s government-guided strategies contributed to the control of the disease’s spread, and thus Taiwan had lower rates of COVID-19 compared with other countries in the beginning of pandemic [3]. Children and adolescents face special challenges based on their life stage, as well as the impact of COVID-19 and subsequent measures aimed at curbing the disease’s contagion and effect on them [4,5]. Although children infected with SARS-CoV-2 have less serious symptoms than adults, some infants or teens still require hospitalization, and the infection can spread through school clusters [6,7]. Therefore, children’s caregivers had psychological distress and serious concerns about the risk of COVID-19 infection [8,9]. Perceptions and attitude of COVID-19 threat might influence caregivers’ protective behaviors (e.g., frequent handwashing, mask wearing, and keeping social distance) toward their children [10].

The most effective way to safely obtain immunity is currently vaccination [11]. Vaccinating all children could be one of the most important public health measures for preventing children or adolescents from being infected with SARS-CoV-2 [12,13]. During the continuous health crisis, parental vaccine hesitancy/delay has been a serious obstacle to vaccination for the youth population [14]. The most common reason reported by caregivers intending to vaccinate was to protect their child, and the most common reason reported by caregivers refusing vaccination was uncertainty about the vaccine’s safety [15]. Vaccine-associated myocarditis was the most severe adverse effect related to COVID-19 vaccination and was thus the main concern of parents’ worry [16,17]. Many teenagers and their caregivers are anxious and hesitant about decision to get vaccinated [18]. To improve parents’ compliance and confidence in vaccine safety, publicity activities ought to provide more information about vaccine safety, especially for young parents who have not yet been vaccinated against COVID-19 [19].

Taiwan Centers for Disease Control announced that children and adolescents between the ages of 12 and 18 could be vaccinated starting in September 2021. However, people remain hesitant to vaccinate teenagers with BNT162b2 (Pfizer–BioNTech) or mRNA-1273 (Moderna) vaccines due to media reports that teenagers are at risk of such symptoms as myocarditis, fever, fatigue, headache, and muscle aches [20,21]. A systematic review revealed that many in the US population have hesitancy, which is highest in Black/African Americans and pregnant or breastfeeding women, but low in the male sex [22]. A survey in Brazil identified that even many hesitant caregivers were willing to vaccinate their offspring against COVID-19 [23]. A study in Ethiopia revealed that full vaccination coverage among children aged 15–23 months remains low [24]. Another study in Greece found that 80% of parents who did not plan to vaccinate their children would vaccinate them if recommended by their pediatrician [25]. Therefore, adequate education and full exploration of parents’ concerns are vital for enhancing the acceptance of the COVID-19 vaccine for children [26].

Caregivers of children in different age groups suffer from different types of anxiety. Understanding a child’s vaccination status and their parents’ attention to COVID-19 may help determine caregivers who are willing to vaccinate their children [27]. Therefore, this study surveyed the attitude, intention, decision-making, and psychological well-being among the caregivers of children who received COVID-19 vaccination in Taiwan. We then compared the above psychological assessments among the caregivers of preschool children, school-age children, and adolescents. In addition, we explored the mental health of caregivers in relation to the COVID-19 pandemic or vaccination-associated factors.

## 2. Methods

### 2.1. Study Design and Measures

Since September 2021, the Taiwan government has authorized children and adolescents to receive the COVID-19 vaccine. This study was a cross-sectional survey. All participants were caregivers of children who received COVID-19 vaccination from 31 March to 30 September 2022. While children were receiving the COVID-19 vaccine in Kaohsiung, their caregivers were invited to fill out a questionnaire. The study covered a period of just over four months (from 30 June to 30 September). Through anonymous data collection, a paper-and-pencil self-test questionnaire was adopted, and online questionnaires were distributed to caregivers via the Internet. The questionnaire consisted of four parts: basic demographic data, Adopting Self-Protective Behavior Scale, Drivers of COVID-19 Vaccination Acceptance Scale (DrVac-COVID19S), Impact of Event Scale (IES), Chinese Health Questionnaire (CHQ), and Parental Bonding Instrument (PBI). The participants were divided into three groups based on the children’s age: preschool (<6 years), school-age (6–12 years), and adolescents (12–18 years).

### 2.2. Adopting Self-Protective Behavior Scale

Using a six-item scale, we determined the frequency of difficulties encountered by caregivers in asking their children to adopt protective behaviors against COVID-19 [28]. The scale was developed based on the recommendations of the Centers for Disease Control and Prevention for protecting against COVID-19 in the general population, including washing hands frequently, wearing masks at all times, avoiding visiting crowded places, and practicing social distancing. This scale also has other items specifically for children: do not touch their mouths, noses, objects, or other people in public places. These items were assessed using a three-point Likert scale. A total score indicates the degree of difficulty that caregivers encounter when asking their children to adopt protective behaviors against COVID-19. According to a previous study, this scale demonstrated high internal consistency and concurrent validity with caregivers’ mental health problems [28].

### 2.3. Drivers of COVID-19 Vaccination Acceptance Scale

The Drivers of COVID-19 Vaccination Acceptance Scale (DrVac-COVID19S) [29] consists of 12 items rated on a 7-point Likert scale from 1 (strongly disagree) to 7 (strongly agree). These 12 items included the following: values (e.g., “It is important that I get the COVID-19 shot”), impact (e.g., “Vaccination is very effective in protecting me from COVID-19”), knowledge (e.g., “I know very well how vaccination protects me from COVID-19”), and autonomy (e.g., “I can choose whether to get the COVID-19 shot”). Each domain has three items; adding up all three items provides the total domain score. A higher score indicates a higher acceptance of the COVID-19 vaccine. A previous study supported the use of the four-factor structure model (i.e., using four CME constructs) of the DrVac-COVID19S [29]. Furthermore, the known-group validity of the DrVac-COVID19S is satisfactory. Measuring the invariance of the DrVac-COVID19S has been indicated [30,31,32].

### 2.4. Impact of Event Scale (IES)

The Impact of Event Scale, a self-report measure, has been used widely in different groups of people and for all kinds of trauma to assess specific stressful life events [33]. All 15 items are rated on a four-point frequency scale (0, not at all; 1, rarely; 3, sometimes; 5, often) for the past week. A higher score represents a greater frequency of intrusive thoughts and attempts at avoidance. The IES has been translated into Chinese and has shown satisfactory validity in a study of oral cancer patients [34]. The IES has also been used to assess adolescent earthquake victims in Taiwan [35].

### 2.5. Chinese Health Questionnaire (CHQ)

The Chinese Health Questionnaire with cultural sensitivity is a self-administered screening instrument used to assess psychiatric morbidity in the ethnic Chinese community [36]. The CHQ was derived from the General Health Questionnaire [37] and has been validated to have satisfactory construct validity and has been applied in a survey of psychiatric morbidity in the community [35] and hospital settings [38]. The CHQ comprises four factors: somatic symptoms; anxiety and worrying; sleep problems; and depression and poor family relationships [39]. We used the 12-item version of CHQ-12 in this study. For example, as used in the community study, the cut-off point of 2/3 was adopted for case/non-case.

### 2.6. Parental Bonding Instrument

We used the traditional Chinese version [40,41] of the Parental Bonding Instrument (PBI)—Parent Version [42] to comprehensively assess parenting behaviors, that is, parental affection/care (12 items, e.g., “I could make the children feel better when they were upset”), parental overprotection (seven items, e.g., “I tried to control everything that the child did”), and authoritarian parenting (six items, e.g., “I let the children do things that they liked to do”). Caregivers rated each item on a 4-point Likert scale ranging from 1 (agree) to 4 (disagree). We reverse-coded the items to facilitate the interpretation process. Caregivers with the highest parental affection/care score were perceived to be affectionate and warm, those with the highest authoritarian parenting score were considered to highly encourage behavioral freedom, and those with a high parental overprotection score were regarded as being devoid of psychological autonomy to a high degree. Studies have shown that the traditional Chinese version of the PBI has satisfactory reliability and validity [40,43].

### 2.7. Statistical Analysis

First, we used a chi-square test or one-way analysis of variance (ANOVA) to compare the perception of the COVID-19 epidemic situation, vaccination consideration, incidence of mental illness, and psychological influence of hospital staff among the three groups (preschool, school-age, and adolescents). The *p*-value in chi-square represented the distribution of categorical variables (e.g., sex) between groups. The *p*-value in one-way ANOVA represented whether there were significant differences in continues variables (e.g., age) between groups. The post-hoc tests were applied to examine the significance of each study group with the other two. Multivariate logistic regression analysis was used to explore the related factors of mental illness in caregivers. The dependent variable was set as psychiatric morbidity (CHQ scores ≥ 3), and the independent variables were children’s and caregivers’ demographic data, COVID-19 Vaccination Acceptance, Impact of Event, and parental bonding. Finally, a stepwise forward model of logistic regression was adopted to test the risk factors associated with psychiatric morbidity (applying likelihood ratio estimation). We used the adjusted odds ratio (aOR) and 95% confidence interval to estimate the risk of psychiatry morbidity of caregivers.

## 3. Results

**Figure 1:**
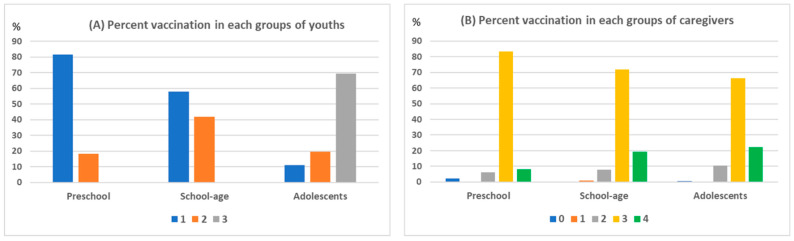
The proportion of vaccination numbers in three age groups of youths (**A**) and their caregivers (**B**).

This study included 443 caregivers of youths who received COVID-19 vaccination (98 preschool children, 191 school-age children, and 154 adolescents) (Table 1). For the youths, sex distribution was not significantly different. The preschool children received either one dose (81.6%) or two doses (18.4%) of the COVID vaccine. The school-age group received either one dose (58.1%) or two doses (41.9%) of the COVID vaccine. The adolescent group received one dose (11.0%), two doses (19.5%), or three doses (69.5%) of the COVID vaccine. Of the caregivers, most responders were children’s mothers (76.5–81.2%). For the caregivers, most of them had received three doses of the COVID-19 vaccine (83.3% in preschool; 71.7% in school-age; 66.4% in the adolescent group), and the caregivers in the adolescent group also showed higher rates of four doses of COVID-19 vaccination (22.4%). Figure 1 illustrates the proportion of vaccination numbers in three age groups of youths and their caregivers.

Table 2 lists the perception, behavioral change, and influence of the COVID-19 pandemic among the caregivers of preschool, school-aged, and adolescent individuals. With regard to the adaptive behaviors to the COVID-19 pandemic, compared to the caregivers of adolescents, the caregivers of preschool children were more likely to avoid going outdoors, maintain indoor ventilation, and wash hands frequently. Furthermore, the intrusion scores (*p* = 0.035) and total scores of the IES (*p* = 0.034) showed significant group differences, and avoidance scores exhibited a marginal group difference (*p* = 0.052). The post-hoc tests revealed that the caregivers of preschool children suffered from more severe symptoms about recurrent, unwanted emotional distress or physical reactions (intrusion, *p* = 0.010), and overall symptoms (total score., *p* = 0.010), compared to the caregivers of adolescents. Moreover, they tried to avoid thinking about, talking, or exposure to the activities or people related to the COVID-19 pandemic (avoidance, *p* = 0.015).

Table 3 demonstrates the perception and intention of COVID-19 vaccination among the caregivers of preschool, school-aged, and adolescent individuals. Over 90% of caregivers (across all age groups) considered that the safety and efficacy of vaccination and whether there was sufficient study done on the COVID-19 vaccination were critical issues. In contrast, among the items regarding perception of vaccine, the costs and duration of vaccination were less important issues for the caregivers to consider. However, there were 51%, 51.3%, and 55.2% among caregivers of preschool, school-age, and adolescents still considered cost as an important issue, respectively. It is interesting to note that the caregivers of preschool children were more concerned about the family’s opinion about the vaccination (78.6%) than those of adolescents (63%) (*p* = 0.026). In terms of vaccination intention, we observed no significant difference in recognition of vaccine’s potential effect (impact), the understanding about how vaccination exerts its protective effect (knowledge), identification of the importance of vaccination (value), or making their own decision about vaccination (autonomy) among the three age groups.

Table 4 lists the mental health and parenting styles of caregivers. Using CHQ (physical symptoms, anxiety and worry, depression, and sleep) to measure the mental health status of caregivers is comparable among the three age groups. In the parental bonding instrument (PBI), we found the protective parenting style (*p* < 0.001) were significant differences among the three age groups. However, there were no significant differences in the perception of being affectionate and warm among groups. In the post hoc tests, we found the caregivers of preschool children showed higher protection tendencies than those of school-age children (*p* = 0.008) and adolescents (*p* < 0.001). In addition, caregivers of school-age children had a greater protective parenting style than those of adolescents (*p* < 0.001).

Table 5 demonstrates the logistic regression model of factors associated with psychiatric morbidity (defined by CHQ) among the caregivers of youths who received COVID-19 vaccination. The multivariate model and stepwise regression model showed consistent results. The intrusion scores of IES (aOR = 1.10, *p* = 0.015) and protection of PBI (aOR = 1.08, *p* < 0.001) were significant factors associated with the psychiatric morbidity of caregivers. This represents that the caregivers who suffered from more severe intrusion symptoms or higher protection tendencies toward their children had greater risk of psychiatric morbidity.

## 4. Discussion

With regard to adaptive behaviors to the COVID-19 pandemic, the caregivers for preschoolers are more likely to prevent going outdoors, maintain indoor ventilation, and wash their hands frequently than the caregivers for adolescents. In addition, the caregivers of preschool children had more stress than those of adolescents and showed a higher protection tendency (PBI). According to a study conducted in Brazil, the caregivers of young children were concerned about providing them with age-appropriate play activities (49.7%), planning the children’s daily routine at home (41.8%), and teaching them when they make a mistake [44]. Psychological symptoms were reported by caregivers following the outbreak of COVID-19 [45]. Once infected with COVID-19, older children may be more vulnerable than younger children, so caregivers may suffer greater stress and be more protective [46].

The Taiwan Centers for Disease Control announced that children and adolescents between the ages of 12 and 18 could get vaccinated starting in September 2021. mRNA-1273 or BNT162b2 vaccinations began for children and adolescents at this time. The majority of caregivers thought that the safety and effectiveness of vaccination, as well as sufficient study on COVID-19 vaccination, were important subjects. However, the costs and duration of vaccination were less important issues for caregivers to consider. According to an international study, after the emergency approval of COVID-19 vaccine for adults, the willingness to speed up approval has increased, but it is still unlikely that mothers will agree to quick approval [47]. Interestingly, the caregivers of preschool children cared more about the family’s opinion about the vaccination (78.6%) than those of adolescents (63%). Again, this phenomenon suggests that, when compared to the caregivers of adolescents, the caregivers of preschool children may be more cautious with regard to deciding whether to vaccinate their children. Therefore, while making decisions for small children, caregivers may rely more significantly on family opinion.

Using CHQ (somatic symptoms, anxiety and worrying, depression, and sleep) to measure the mental health status of caregivers was comparable among the three age groups. Nevertheless, the intrusion scores of IES and protection of PBI were significant factors related to the incidence of mental illness among caregivers. Higher epidemic pressure was related to the lower confidence of caregivers to meet their family’s COVID-19 needs, which correlated with worse caregiver mental health symptoms [45]. The caregivers who have great pressure from COVID-19 and tend to their children have lower resilience to the subsequent psychological impact and have poor mental health. Therefore, this kind of caregiver guarantees the further policy implementation of vaccinated youths in the future [48].

This study has certain strengths. It is the first to compare the perspectives of vaccination in COVID-19 among the caregivers of preschool children, school-age children, and adolescents. Second, we comprehensively investigated the intention, attitude, and psychiatric morbidity of the caregivers in this study. However, this study also has certain limitations that need to be considered when interpreting the data. First, the sample size was not large. Furthermore, participants were recruited from communities in one large city in Southern Taiwan, and the sample may not represent the caregivers of children in Taiwan. Second, this study is a cross-sectional survey. The participants had different time points to complete the survey. The questionnaires were filled out by some participants after their children received the first, second, or third doses of vaccines. In addition, the COVID-19 pandemic in Taiwan waxed and waned [49,50]. Therefore, the impact of the COVID-19 pandemic or the prospect of vaccination may also have gone up and down. Third, this study used a self-administered questionnaire, and the lack of objective assessment (e.g., diagnostic interviews) may have led to biased results.

## 5. Conclusions

In this study, the COVID-19 vaccination perspective was compared among the caregivers of preschool children, school-age children, and adolescents. Compared to the caregivers of adolescents, the caregivers of preschool children exhibited higher protective responses toward the COVID-19 pandemic. Furthermore, the caregivers of preschool children had a greater higher emotional impact (IES) than those of adolescents, and they were more concerned with their families’ reactions to vaccinations. Finally, we found that thought intrusion of COVID-19 and a protective parenting style were significantly associated with the psychiatric morbidity of caregivers. These results may serve as an important reference for health care and policy-making regarding COVID-19 vaccination for youths.

## Figures and Tables

**Table 1:** Characteristics of preschool, school-aged and adolescents individuals and their caregivers.

	Preschool (*n* = 98)	School-Age (*n* = 191)	Adolescents (*n* = 154)	X or F	*p*-Value
Youths					
Sex (male)	49 (50%)	92 (48.2%)	75 (48.7%)	0.087	0.957
Age (years, mean ± SD)	4.7 ± 0.6	8.1 ± 1.5	14.0 ± 1.8	1306.3	<0.001 *
Mental illness history	4 (4.1%)	9 (4.7%)	7 (4.5%)	0.088	1.000
How many shots of COVID vaccine				292.9	<0.001 *
1	80 (81.6%)	111 (58.1%)	17 (11.0%)		
2	18 (18.4%)	80 (41.9%)	30 (19.5%)		
3	0 (0%)	0 (0%)	107 (69.5%)		
Caregivers					
Sex (male)	23 (23.5%)	44 (23.0%)	29 (18.8%)	1.128	0.569
Age (years, mean ± SD)	39.5 ± 5.7	41.7 ± 4.5	45.9 ± 4.7	58.5	<0.001 *
Numbers of children				8.074	0.089
1	28 (28.6%)	36 (18.8%)	22 (14.3%)		
2	55 (56.1%)	119 (62.3%)	100 (64.9%)		
≥3	15 (15.3%)	36 (18.8%)	32 (20.8%)		
Education				0.362	0.835
Senior high school or lower	20 (20.4%)	36 (18.8%)	33 (21.4%)		
College or above	78 (79.6%)	155 (81.2%)	121 (78.6%)		
Marriage status				0.663	0.718
Married and cohabitation	89 (90.8%)	171 (89.5%)	135 (87.7%)		
Separated or divorced	9 (9.2%)	20 (10.5%)	19 (12.3%)		
How many shots of COVID vaccine				16.338	0.014 *
0	2 (2.1%)	0 (0%)	1 (0.7%)		
1	0 (0%)	2 (1.0%)	0 (0%)		
2	6 (6.3%)	15 (7.9%)	16 (10.5%)		
3	80 (83.3%)	137 (71.7%)	101 (66.4%)		
4	8 (8.3%)	37 (19.4%)	34 (22.4%)		
Ideal option of COVID vaccine for child				11.015	0.201
BNT	76 (77.6%)	133 (69.6%)	100 (64.9%)		
Moderna	8 (8.2%)	27 (14.1%)	17 (11.0%)		
AZ	0 (0%)	1 (0.5%)	0 (0%)		
MVC	2 (2.0%)	7 (3.7%)	12 (7.8%)		
Any will be fine	12 (12.2%)	23 (12.0%)	25 (16.2%)		

AZ: Oxford–AstraZeneca COVID-19 vaccine; BNT: BNT162b2 (Pfizer–BioNTech); Moderna: mRNA-1273; MCV: Medigen Vaccine Biologics Corporation (MVC)-COV1901. X and F was estimated using chi-square test and one-way analysis of variance (ANOVA), respectively. * *p* < 0.05.

**Table 2:** The perception, behavioral change and influence of COVID-19 pandemic among the caregivers of preschool, school-aged and adolescents individuals.

	Preschool (*n* = 98)	School-Age (*n* = 191)	Adolescents (*n* = 154)	X or F	*p*-Value
Adaptive behaviors					
Avoid going outside	83 (84.7%)	159 (83.2%)	110 (71.4%)	9.41	0.009 *
Maintain good indoor ventilation	79 (80.6%)	126 (66.0%)	89 (57.8%)	14.00	0.001 *
Cleaning or sanitizing the residence	59 (60.2%)	109 (57.1%)	77 (50.0%)	2.945	0.229
Washing hands often	85 (86.7%)	151 (79.1%)	109 (70.8%)	9.12	0.010 *
Wearing a mask consistently	89 (90.8%)	162 (84.8%)	129 (83.8%)	2.695	0.260
Impact of Event Scale (IES)					
Intrusion	13.9 ± 5.5	12.3 ± 4.5	12.7 ± 5.3	3.37	0.035 *^,a^
Avoidance	15.3 ± 5.7	13.7 ± 4.9	14.2 ± 5.7	2.98	0.052 ^b^
Total score	29.2 ± 10.7	26.0 ± 9.1	26.9 ± 10.7	3.41	0.034 *^,c^

* *p* < 0.05. Post-hoc tests: ^a^ P (preschool) > S (school-age), *p* = 0.010; ^b^ P > S, *p* = 0.015; ^c^ P > S, *p* = 0.010.

**Table 3:** The perception, and intention of COVID-19 vaccination among the caregivers of preschool, school-aged and adolescents individuals.

	Preschool (*n* = 98)	School-Age (*n* = 191)	Adolescents (*n* = 154)	X or F	*p*-Value
Perception of Vaccine					
Safety	96 (98.0%)	190 (99.5%)	153 (99.4%)	1.836	0.399
Efficacy	97 (99.0%)	189 (99.0%)	153 (99.4%)	0.17	0.918
Cost	50 (51.0%)	98 (51.3%)	85 (55.2%)	0.642	0.726
Duration of vaccination	55 (56.1%)	100 (52.4%)	76 (49.4%)	1.107	0.575
Sufficient research	97 (99.0%)	188 (98.4%)	153 (99.4%)	0.662	0.718
Family’s opinion	77 (78.6%)	137 (71.7%)	97 (63.0%)	7.33	0.026 *
Intention of vaccinated					
Impact	6.4 ± 2.8	6.8 ± 3.3	6.6 ± 3.3	0.437	0.646
Knowledge	6.5 ± 2.7	6.8 ± 2.6	7.0 ± 2.5	1.371	0.255
Value	5.8 ± 2.8	6.3 ± 3.2	6.3 ± 3.2	1.043	0.353
Autonomy	6.8 ± 3.0	7.2 ± 3.1	7.5 ± 2.8	1.445	0.237

* *p* < 0.05.

**Table 4:** The mental health and parenting style among the caregivers of preschool, school-aged and adolescents individuals.

	Preschool (*n* = 98)	School-Age (*n* = 191)	Adolescents (*n* = 154)	X or F	*p*-Value
Chinese Health Questionnaire (CHQ)					
Somatic symptoms	7.0 ± 2.8	6.7 ± 2.4	6.3 ± 2.2	2.62	0.074
Anxiety and worrying	6.1 ± 2.1	5.7 ± 1.9	5.7 ± 2.0	1.55	0.21
Depression	7.5 ± 1.7	7.1 ± 1.8	7.2 ± 1.8	1.953	0.143
Sleep	1.9 ± 0.8	1.8 ± 0.7	1.9 ± 0.8	0.578	0.562
Parental Bonding Instrument (PBI)					
Affection/Care	38.7 ± 4.8	39.5 ± 4.5	39.1 ± 5.7	0.865	0.422
Protection	27.1 ± 4.3	25.5 ± 4.7	23.4 ± 5.5	17.24	<0.001 *^,a^

* *p* < 0.05. Post-hoc tests: ^a^ P (preschool) > S (school-age): *p* = 0.008; P > A (adolescent): *p* < 0.001; S > A: *p* < 0.001.

**Table 5:** Logistic regression of factors associated with psychiatry morbidity among the caregivers of youths who received COVID-19 vaccination.

	Multivariate Model		Backward Stepwise	
Variables	β (95% CI)	*p*-value	β (95% CI)	*p*-value
Child’s age	0.99 (0.93–1.06)	0.751		
Child’s sex (F vs. M)	1.12 (0.74–1.70)	0.598		
Caregiver’s age	1.00 (0.96–1.05)	0.867		
Caregiver’s sex (F vs. M)	0.97 (0.58–1.61)	0.894		
Caregiver’s education (high vs. low)	1.02 (0.60–1.72)	0.941		
Intention of vaccinated				
Impact	1.04 (0.91–1.19)	0.542		
Knowledge	0.97 (0.84–1.11)	0.612		
Value	1.04 (0.90–1.19)	0.633		
Autonomy	0.97 (0.88–1.07)	0.554		
Impact of Event Scale (IES)				
Intrusion	1.10 (1.02–1.19)	0.015 *	1.13 (1.08–1.17)	<0.001 *
Avoidance	1.02 (0.95–1.10)	0.627		
Parental Bonding Instrument (PBI)				
Affection/Care	0.97 (0.93–1.01)	0.177		
Protection	1.08 (1.02–1.13)	0.004 *	1.09 (1.05–1.14)	<0.001 *

F: female; M: male. * *p* < 0.05.

## Data Availability

The data will be available upon reasonable request to the corresponding authors.
